# Total Flavones of* Rhododendron simsii* Planch Flower Protect against Cerebral Ischemia-Reperfusion Injury via the Mechanism of Cystathionine-*γ*-Lyase-Produced H_2_S

**DOI:** 10.1155/2018/8903849

**Published:** 2018-05-31

**Authors:** Shuo Chen, Jian-Hua Zhang, You-Yang Hu, Dong-Hua Hu, Shan-Shan Gao, Yi-Fei Fan, Yu-Ling Wang, Yi Jiao, Zhi-Wu Chen

**Affiliations:** ^1^Department of Physiology, Anhui Medical University, Hefei, Anhui 230032, China; ^2^Department of Pharmacology, Anhui Medical University, Hefei, Anhui 230032, China; ^3^Department of Cardiology, First Affiliated Hospital of Jinan University, Guangzhou, Guangdong 510630, China; ^4^Department of Anesthesiology, Anhui Chest Hospital, Hefei, Anhui 230032, China

## Abstract

Total flavones of* Rhododendron simsii *Planch flower (TFR) have a significant protective effect against cerebral ischemia-reperfusion injury. However, its mechanism is unclear. This study investigated the protection of TFR against cerebral ischemia-reperfusion injury via cystathionine-*γ*-lyase- (CSE-) produced H_2_S mechanism. CSE^−/−^ mice and CSE-siRNA-transfected rat were used. Relaxation of cerebral basilar artery (CBA), H_2_S, and CSE mRNA were measured. TFR significantly inhibited cerebral ischemia-reperfusion-induced abnormal neurological symptom and cerebral infarct in the normal rats and the CSE^+/+^ mice, but not in the CSE^−/−^ mice, and the inhibition was markedly attenuated in CSE-siRNA-transfected rat; TFR elicited a significant vasorelaxation in rat CBA, and the relaxation was markedly attenuated by removal of endothelium or CSE-siRNA transfection or coapplication of NO synthase inhibitor L-NAME and PGI_2_ synthase inhibitor Indo. CSE inhibitor PPG drastically inhibited TFR-evoked vasodilatation resistant to L-NAME and Indo in endothelium-intact rat CBA. TFR significantly increased CSE mRNA expression in rat CBA endothelial cells and H_2_S production in rat endothelium-intact CBA. The increase of H_2_S production resistant to L-NAME and Indo was abolished by PPG. Our data indicate that TFR has a protective effect against the cerebral ischemia-reperfusion injury via CSE-produced H_2_S and endothelial NO and/or PGI_2_ to relax the cerebral artery.

## 1. Introduction

Cerebral ischemia-reperfusion (I/R) injury occurs when blood supply returns following brain ischemia such as acute ischemic stroke, and it is one of the most common pathologies responsible for death and acquired disability in adults worldwide. Despite the pathogenic mechanism of cerebral I/R injury has not been fully elucidated, it is generally believed that cerebral vascular endothelium is involved in the process underlying the pathogenesis.

Vascular endothelium is crucial in maintaining an adequate vascular tone and organ blood flow by releasing a variety of endothelium-derived relaxing factors (EDRFs). Therefore, the endothelium plays an important role in the pathological process of cerebral I/R injury. EDRFs include endothelial nitric oxide (NO) and prostacyclin (PGI_2_) as well as endothelium-dependent hyperpolarizing factor (EDHF) [1]. Endothelial NO and PGI_2_ could, respectively, activate soluble guanylate cyclase and inositol phosphate receptor in vascular smooth muscle cell (VSMC), resulting in vasodilation. EDHF could induce a brief hyperpolarization of VSMC and a subsequent vasodilatation, which is resistant to NO synthase inhibitor and PGI_2_ synthase inhibitor [2, 3]. Thus, EDHF response is characterized as an endothelium-derived non-NO and non-PGI_2_ (non-NO/PGI_2_) factor-mediated hyperpolarization and vasorelaxation. The EDHF response is presented in a wide variety of arteries from different species including humans [4]. In peripheral arteries such as the mesenteric artery and coronary, several candidate factors have been proposed as EDHF, including epoxyeicosatrienoic acids (EETs) derived from cytochrome P_450_ [5] and hydrogen peroxide (H_2_O_2_) [6]. However, the identity of EDHF in cerebral blood vessels is likely different from that in peripheral arteries. It has been noted that EDHF-induced dilation in rat cerebral artery does not involve EETs or H_2_O_2_ [7]. Our previous study also demonstrated that neither EETs nor H_2_O_2_ mediated EDHF-induced dilation in rat cerebral basilar artery (CBA) [8], a main artery supplying the cerebellum, brain stem, and other encephalic regions cerebellum.

Recently, hydrogen sulfide (H_2_S) has been identified as a novel signaling molecule and received an increasing attention as an endogenous vasodilator [9]. It was pointed out that H_2_S is a dilator of the cerebral circulation in newborn pigs [10]. The endogenous H_2_S is physiologically generated from L-cysteine by H_2_S generating-enzyme. Cystathionine-*γ*-lyase (CSE), a specific enzyme responsible for endogenous H_2_S production in vascular tissues, can produce abundant H_2_S to decrease vascular tone [10]. In addition, H_2_S-induced endothelium-derived relaxation of rat mesenteric artery was shown to be inhibited by the blockade of calcium-activated potassium (K_Ca_) channels [11]. Our recent studies also indicated that H_2_S shares common traits of EDHF in rat CBA and the middle cerebral artery (MCA), suggesting that it could act as an EDHF in rat cerebral arteries [12–15].

*Rhododendron simsii *Planch flower, a Chinese herbal medicine, has been used in China for thousands of years to treat various diseases.* Rhododendron simsii *Planch flower has a therapeutic effect on ischemic cerebral apoplexy, and its main effective ingredients are flavones. As we all know that flavones, derived from either 2-phenyl-benzopyrone or 3-phenyl-benzopyrone, are widely distributed in natural plants and have important effects in the regulation of physiological functions. Total flavones of* Rhododendron simsii *Planch flower (TFR) are an effective part extracted from this flower and are primarily comprised of hyperin, quercetin, rutin, and other flavone glycosides [16]. TFR has a significant protective effect against cerebral I/R injury [17]. However, the protective mechanism remains poorly understood. It was reported that some flavones could dilate blood vessel via an EDHF-mediated mechanism [18, 19]. Our previous study [15] shows that endogenous H_2_S is a component of the EDHF response of rat CBA induced by hyperin, one of main effective components of TFR. By using CSE gene knockout (CSE KO) mice, the small double-stranded interfering RNA (siRNA) technique and other methods, the present study was therefore designed to test the hypothesis that endogenous CSE-produced H_2_S participates in protective effect of TFR against the cerebral I/R injury via cerebral vasodilatation and to focus on the role of endothelial CSE-produced H_2_S in EDHF-mediated relaxation of rat CBA to TFR.

## 2. Materials and Methods

### 2.1. Animals

Adult CSE^+/+^ and CSE^−/−^ C57BL/6J mice (with 20~24 g body weight, aged 12~15 weeks, female to male = 1 : 1) were provided by Shanghai Biomodel Organism Science & Technology Development Co., Ltd.; male Sprague-Dawley rats weighing 250~350 g were purchased from the Experimental Animal Center of Anhui Medical University. The rats were housed in transparent plexiglas cages with stainless steel wire lids and filter tops under a 12 h light/dark cycle in a temperature (20–23°C) and humidity (60 ± 10%) controlled room. Food and water were available ad libitum. All animal procedures for this investigation were approved by Anhui Medical University Animal Care Committee and are in compliance with the Guide for the Care and Use of Laboratory Animals published by the US National Institutes of Health (Publication No. 85-23, revised 2011).

### 2.2. Chemicals and Solutions

TFR was provided by Hefei Heyuan Medicine Technology Co., Ltd. (Hefei, China); the content of flavones was greater than 85%; 9,11-dideoxy-11*α*, 9*α*-epoxymethano-prostaglandin F_2*α*_ (U_46619_), NG-nitro-L-arginine methyl ester (L-NAME), indomethacin (Indo), DL-propargylglycine (PPG), 2,3,5-triphenyltetrazolium chloride (TTC), and ACh were purchased from Sigma (St. Louis, USA); RT-PCR test kit was purchased from Dalian Baoshengwu Biological Co., Ltd. (Dalian, China); CSE-siRNA was purchased from GenePharma (Shanghai, China); Atelocollagen was purchased from KOKEN (Tokyo, Japan). Phosphate saline solution (PSS, adjusted pH to 7.4 with NaOH) comprised the following (mM): NaCl 118, KCl 3.4, CaCl_2_ 2.5, KH_2_PO_4_ 1.2, MgSO_4_ 1.2, NaHCO_3_ 25, and glucose 11.1, and the solution was bubbled with 95% O_2_ and 5% CO_2_.

### 2.3. Mouse Cerebral I/R Model

Mouse cerebral I/R injury was induced by the method of MCA occlusion (MCAO) [17] under anesthesia by 3.5% chloral hydrate peritoneal injection (300 mg/kg) and fixed on a heated operation table to maintain body temperature at 37.5 ± 0.5°C. The right common and external carotid arteries (CCA and ECA) and internal carotid arteries (ICA) were, respectively, isolated through a midline neck incision. After the ECA being ligated, a fish thread at a diameter of 0.185 mm with a round tip was gently introduced into the ICA via the CCA until it passed the MCA origin (approximately 15 mm) to occlude the MCA. After 2 h of occlusion, the thread was withdrawn and the MCAO territory was reperfused. In the sham group, the thread did not reach the MCA origin (no more than 10 mm) so that the MCA was not occluded. Mouse body temperature was maintained at 37°C during the experiment. TFR 100 mg/kg was administrated by intravenous injection at 30 min before MCAO.

At 24 h of the reperfusion, the examination of abnormal neurological symptom in mouse was performed according to an established scoring system. Briefly, the mouse had no sign of neurological disorder, scaled 0; flex and adduction of the contralateral limbs when mouse tail being lifted, scaled 1; muscle resistance was weakened when mouse being pushed to the right side, scaled 2; rotation to the contralateral side when mouse crawling, scaled 3; mouse without spontaneous activities or unconscious, scaled 4.

After neurological assessment, mouse received an overdose of chloral hydrate. Brain was harvested and sliced into 2 mm thick coronal sections. The slices were incubated in 2% TTC solution at 37°C for 30 min in the dark. Normal brain tissue was stained orange red, and infarct area was stained white. The slices were fixed with 4% poly formaldehyde. All slices from one brain were weighed. Then the white infarction tissues in the slices were carefully separated. Percentage of cerebral infarct was calculated by weight of infracted tissues and weight of all slices from one brain.

### 2.4. CSE-siRN Transfection in Rat In Vivo

CSE-siRNA-transfected rat was prepared as previously described [14]. Briefly, rats were randomly divided into control group and CSE-siRNA transfection group. After rats were anesthetized with 10% chloral hydrate by intraperitoneal injection, the right CCA and ECA were isolated via a ventral midline incision, and the ECA was ligated. Then, rats in the control group and the CSE-siRNA transfection group were, respectively, injected with 100 *μ*l atelocollagen + 100 *μ*l physiological saline and 100 *μ*l atelocollagen + 1 OD CSE-siRNA 100 *μ*l through the CCA along the direction of blood flow. The sequence of CSE-siRNA forward was 5′-GGU UAU UUA UCC UGG GCU GTT-3′ and the reverse was 5′-CAG CCC AGG CUA AAU AAC CTT-3′.

At 48 h after CSE-siRNA transfection, the rats were used for the cerebral I/R experiment and the vessel experiment.

### 2.5. Rat Cerebral I/R Model

The experimental procedure was identical to the aforementioned mouse cerebral I/R model. Rat was subjected to 2 h of MCAO followed by 24 h of reperfusion. However, the rat was anesthetized with 10% chloral hydrate peritoneal injection (300 mg/kg). The thread diameter was about 0.235 mm, and the thread was inserted from the right CCA to the right ICA until it passed the MCA origin (approximately 20 mm). At the end of the experiment, score of rat abnormal neurological symptom and percentage of rat cerebral infarct were, respectively, evaluated by using the above methods.

In order to exclude difference of cerebral I/R injury levels between the control rat and the CSE-siRNA-transfected rat, inhibitory percentage was used to evaluate effect of TFR on rat neurological symptom and cerebral infarct. Inhibitory percentage was calculated using the following formula:

(1) $$\begin{array}{c} Inhibitory\;\;percentage\left(\;\%\;\right)=\frac{\left(A-B\right)}{A}\times 100\%, \end{array}$$

where *A* is the average score of neurological symptoms or the average percentage of cerebral infarcts of rats in the I/R group; *B* is the score of neurological symptom or the percentage of cerebral infarct of individual rat treated with TFR.

### 2.6. Vessel Experiment

Rats were anesthetized with 10% chloral hydrate by peritoneal injection and killed humanely by cervical dislocation. The brain was rapidly removed and placed in refrigerated PSS. CBA was then carefully isolated from the brain and cut into segments of 3 mm in length. As previously described [14, 15, 19, 20], with the aid of a dissecting microscope the CBA segment was cannulated at both ends with glass micropipettes, secured with nylon monofilament suture, and placed in an experimental chamber, which was perfused with PSS aerated with 95% O_2_ + 5% CO_2_, and kept at 37°C. The chamber was then placed on the stage of an inverted microscope connected to a digital imaging system (Nikon). The CBA segment was maintained at a constant transmural pressure of 85 mmHg by raising PPS reservoirs connected to the micropipettes to the appropriate height above the vessel. By setting the inflow and outflow rate the luminal flow was adjusted to 150 *μ*l/min. After 1 h equilibrium, 100 nM U_46619_ was added to the luminal superfusate until repeatability contractions were obtained. The CBA image was projected on a video monitor, and the internal diameter was continuously determined by a video dimension analyzer with the data acquisition system. Changes in vessel diameter were observed after application of different treatments.

The CBA dilatation was expressed as the percentage of the maximum diameter (%  *D*_max_) using the following formula:

(2) $$\begin{array}{c} Relaxation\;\;\left(\;\%\;\right)=\frac{\left(D_{x}-D_{min}\right)}{\left(D_{max}-D_{min}\right)}. \end{array}$$

In the above formula, *D*_max_ is the initial diameter after 1 h equilibrium at a constant transmural pressure of 85 mmHg, *D*_min_ is the stable diameter of CBA preconstracted by 100 nM U_46619_, and *D*_*x*_ is the diameter after administration of each concentration of TFR.

In order to examine endothelial dependence of relaxation, the endothelium was mechanically removed by rubbing the luminal surface of the segment with a human hair, and the functional removal of the endothelium was confirmed by the lack of a relaxant response to ACh in the beginning of each experiment.

### 2.7. Determination of H_*2*_S in the Luminal Perfusate

The luminal perfusate sample was collected after the vessel experiment. The H_2_S concentration was measured with the method previously described [14]. Briefly, 0.1 mL perfusate sample was mixed with 0.5 mL 1% acetic acid zinc and 2.5 mL distilled water, and the solution was kept at room temperature for 10 min. By adding in 1 ml of 10% trichloroacetic acid, the solution was centrifugated (14,000*g*) for 10 min to remove the protein in the sample. Then, 0.4 mL of 30 mmol/L FeCl_3_ in 1.2 mol/L HCl and 0.5 mL of 20 mmol/L N,N-dimethyl-p-phenylenediamine dihydrochloride in 7.2 mol/L HCl were added to the supernatant, and the solution was incubated at room temperature for 20 min. The optical absorbance value of the solution was detected at 665 nm with a spectrophotometer. The H_2_S concentration in each sample was calculated against the calibration curve of the standard H_2_S solution.

### 2.8. Reverse Transcriptase Polymerase Chain Reaction (RT-PCR) Method

Primary cultures of rat CBA endothelial cells were prepared similarly as previously described [21]. Three-week-old rats were anesthetized with 10% chloral hydrate by intraperitoneal injection and humanely decapitated. Under sterile conditions, the brain was quickly harvested, and the CBA was carefully removed and cut into segments of 2~3 mm. The artery segment was turned within and outside the flip, so as to put the endothelium being in the external side. By ligation of both ends, the segment was placed in 1% gelatin-coated plastic culture dish added the culture medium DMEM (containing penicillin, streptomycin, glutamine, and vascular endothelial growth factor) under 5% CO_2_ + 95% air atmosphere at 37°C. When the cultures reached 80% confluency (8th day in vitro), TFR or vehicle was added to the culture medium for 12 h. Then, the cultured cells were collected and identified by immunofluorescence staining of factor VIII.

CSE mRNA was determined by RT-PCR method [17]. Total RNA was extracted with RNA isolation kit according to the manufacturer's protocol. The forward primer was 5′-CCACCACAACGATTACCCA-3′; the reverse primer was 5′-TCAGCACCCAGAGCCAAAG-3′. The amplified fragment corresponds to bp 334 of rat CSE (AY032875). 35 cycles; 94°C, 2 minutes; 50°C, 30 secs; 72°C, 45 secs. *β*-actin was used for an internal control.

### 2.9. Statistics

The data were expressed as means ± standard deviation. Statistical analysis was performed with SPSS 11.5. One-way analysis of variance (ANOVA) with LSD post hoc test was used for comparisons between groups. A value of *P* < 0.05 was considered as statistically significant.

## 3. Results

### 3.1. Effect of CSE KO on Protection of TFR on Mouse Cerebral I/R Injury

**Figure 1:**
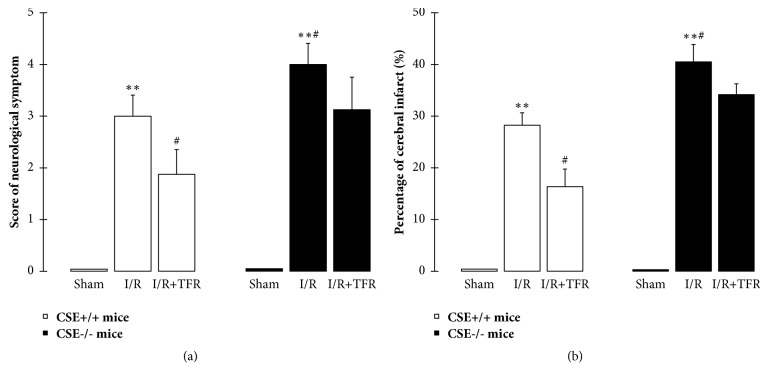
Effect of cystathionine-*γ*-lyase gene knockout on the protection of total flavones of* Rhododendron simsii *Planch flower (TFR) against cerebral ischemia-reperfusion (I/R) injury in mice (mean ± SD, *n* = 6). (a) Neurological symptom. (b) Cerebral infarct. 100 mg/kg TFR was injected intravenously at 30 min before ischemia. ^*∗∗*^*P* < 0.01 versus the sham group; ^#^*P* < 0.05 versus the I/R (CSE^+/+^ mice) group.

Figure 1 showed that compared with the sham group, 2 h of MCAO followed by 24 h of reperfusion induced a significant cerebral I/R injury indicated by an abnormal neurological symptom and cerebral infarct occurred in both CSE^+/+^ mice and CSE^−/−^ mice (*P* < 0.01). The cerebral I/R-induced neurological symptom and cerebral infarct markedly increased in CSE^−/−^ mice compared with those in CSE^+/+^ mice (*P* < 0.05). 100 mg/kg TFR significantly alleviated the neurological symptom and cerebral infarct in CSE^+/+^ mice (*P* < 0.05), but not in CSE^−/−^ mice. The results indicated that CSE KO aggravated the cerebral I/R injury in mice, and CSE was involved in the protection of TFR from cerebral I/R injury.

### 3.2. Effect of CSE Knockdown on Protection of TFR on Rat Cerebral I/R Injury

Our previous study [14] indicates that CSE-siRNA transfection in rat in vivo was effective in the knockdown of CSE expression and H_2_S production. The present study thus prepared the CSE-siRNA-transfected rat to study role of H_2_S in protective effect of TFR on cerebral I/R injury.

#### 3.2.1. Effect of CSE-siRNA Transfection on Cerebral I/R Injury in Rat

**Figure 2:**
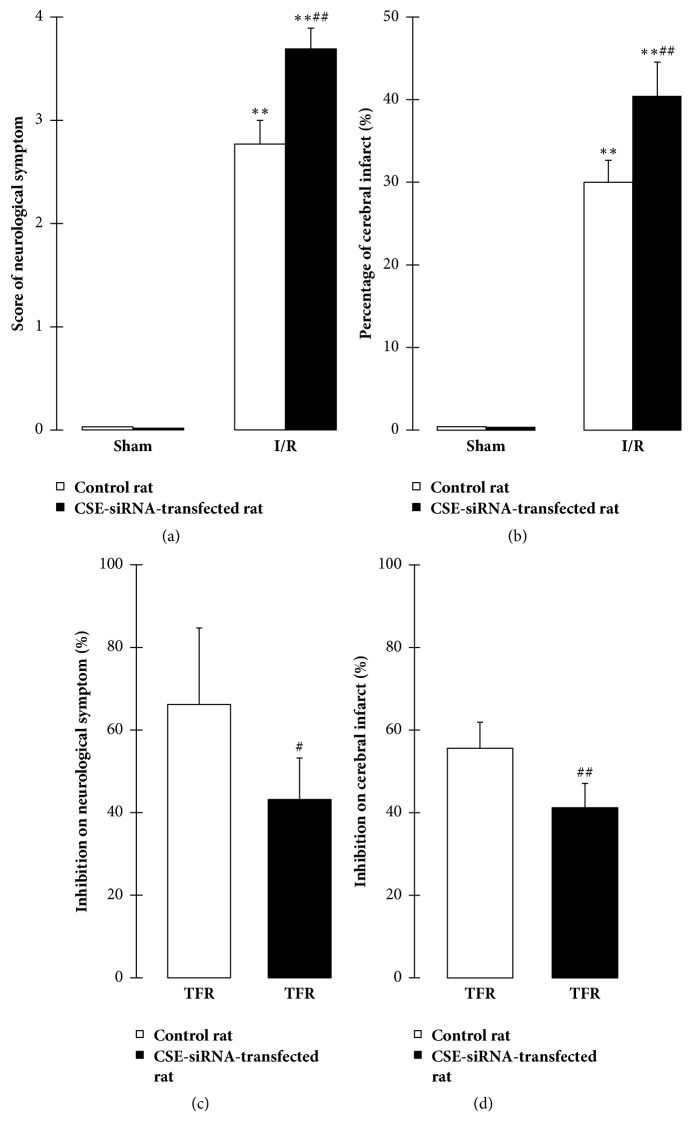
Effect of CSE-siRNA transfection in vivo on the protection of total flavones of* Rhododendron simsii *Planch flower (TFR) against cerebral ischemia-reperfusion (I/R) injury in rat (mean ± SD, *n* = 6). (a) Effect of the transfection on the neurological symptom. (b) Effect of the transfection on the cerebral infarct. (c) Effect of the transfection on TFR-inhibited the neurological symptom. (d) Effect of the transfection on TFR-inhibited the cerebral infarct. 100 mg/kg TFR was administered by intravenous injection at 30 min before ischemia. ^*∗∗*^*P* < 0.01 versus the sham group; ^#^*P* < 0.05, ^##^*P* < 0.01 versus the control rat.

As shown in Figures 2(a) and 2(b), compared with the sham group, cerebral I/R caused abnormal neurological symptom and cerebral infarct in both the control rat and the CSE-siRNA-transfected rat (*P* < 0.01). However, the neurological symptom and cerebral infarct in the CSE-siRNA-transfected rat were significantly aggravated compared to those in the control rat (*P* < 0.01). The results demonstrated that the CSE knockdown resulted in an obvious aggravation of cerebral I/R injury in rat.

#### 3.2.2. Effect of CSE-siRNA Transfection on Inhibition of TFR on Rat Cerebral I/R Injury

Figures 2(c) and 2(d) showed that inhibition of TFR on cerebral I/R-induced neurological symptom and cerebral infarct in the CSE-siRNA-transfected rat were significantly weaker than those in the control rat (*P* < 0.05 or *P* < 0.01); the result indicated that CSE knockdown could attenuate the protective effect of TFR against cerebral I/R injury in rat.

### 3.3. TFR-Induced Relaxation in Rat CBA

The expression of CSE is tissue-special [10]. In brain tissue, CSE only express in cerebrovasculature. Cerebral artery was therefore used to investigate the role of CSE and CSE-produced H_2_S in the protective effect of TFR against cerebral I/R injury.

#### 3.3.1. TFR-Induced Relaxation in Both Endothelium-Intact and Endothelium-Denuded Rat CBA

**Figure 3:**
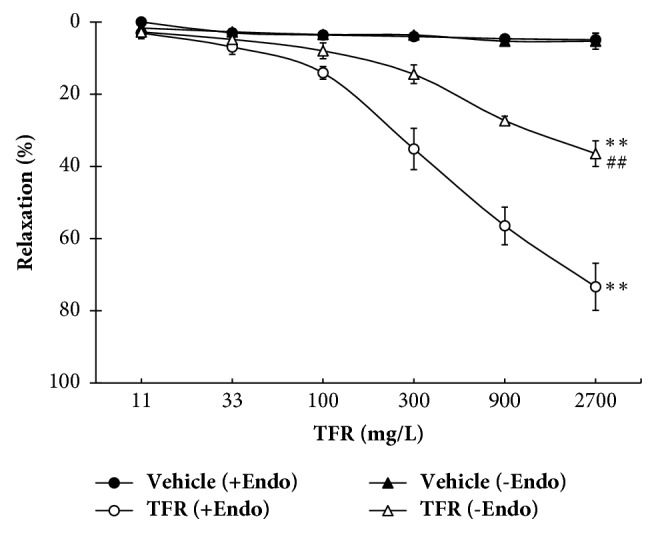
Concentration-response curves for total flavones of* Rhododendron simsii *Planch flower- (TFR-) induced vasorelaxation in endothelium-intact (+Endo) and -denuded (−Endo) rat cerebral basilar artery (mean ± SD, *n* = 6). ^*∗∗*^*P* < 0.01 versus the vehicle group; ^##^*P* < 0.01 versus the TFR (+Endo) group.

As shown in Figure 3, vasorelaxation of TFR (11 to 2700 mg/L) was examined in both endothelium-intact and endothelium-denuded rat CBA precontracted with 100 nM U_46619_. Compared with vehicle, TFR induced a significant and concentration-dependent relaxation of endothelium-intact rat CBA with *E*_max_ of 73.4 ± 6.5% (*P* < 0.01). TFR-induced dilation in endothelium-denuded rat CBA was also marked (*P* < 0.01), but compared with relaxation of TFR in endothelium-intact rat CBA, the relaxation was significantly attenuated (*P* < 0.01), with *E*_max_ being reduced to 32.8 ± 3.5%. No relaxant response was observed in vehicle-treated endothelium-intact or endothelium-denuded rat CBA.

#### 3.3.2. Change of TFR-Induced Relaxation in Rat CBA from the CSE-siRNA-Transfected Rat

**Figure 4:**
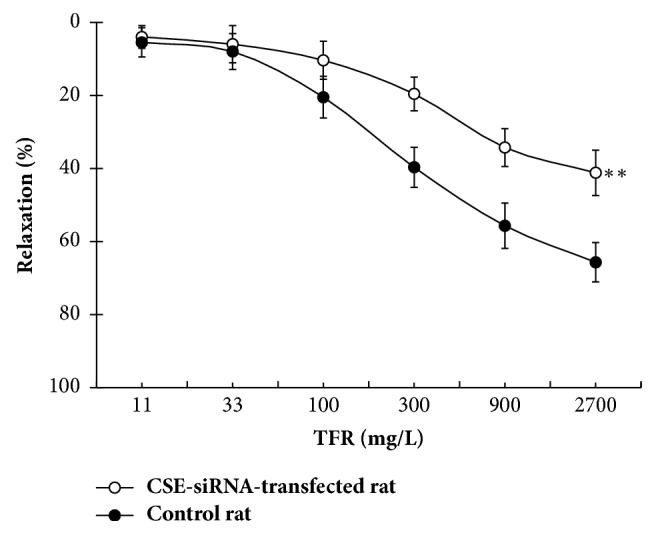
Effect of CSE-siRNA transfection in vivo on the TFR-induced relaxation in rat cerebral basilar artery (mean ± SD, *n* = 6). ^*∗∗*^*P* < 0.01 versus the control rat.

As shown in Figure 4, TFR-induced relaxation was markedly attenuated in endothelium-intact CBA from the CSE-siRNA-transfected rat compared to that from control rat (*P* < 0.01); the result indicated that CSE was involved in the cerebral relaxation of TFR.

### 3.4. Effect of L-NAME + Indo on TFR-Induced Relaxation in Endothelium-Intact Rat CBA

**Figure 5:**
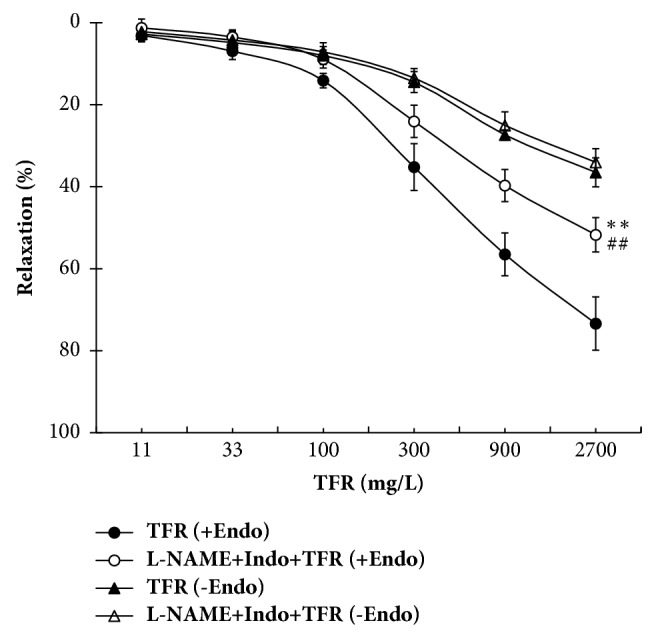
Combinative effect of nitric oxide synthase inhibitor L-NAME (30 *μ*M) and cyclooxygenase inhibitor Indo (10 *μ*M) on total flavones of* Rhododendron simsii *Planch flower- (TFR-) induced relaxation in endothelium-intact (+Endo) and -denuded (−Endo) rat cerebral basilar artery (mean ± SD, *n* = 6). ^*∗∗*^*P* < 0.01 versus the TFR (+Endo) group; ^##^*P* < 0.01 versus the TFR (−Endo) group.

The attenuation of TFR-induced relaxation after removal of the endothelium suggests a contribution of EDRFs. The roles of NO and PGI_2_ in the relaxation were further studied. As shown in Figure 5, a combination of NO synthase inhibitor L-NAME (30 *μ*M) and cyclooxygenase inhibitor Indo (10 *μ*M) significantly attenuated the vasorelaxation of rat CBA with endothelium to TFR (*P* < 0.01); *E*_max_ was reduced from 73.4 ± 6.5% in the absence of L-NAME + Indo [the TFR (+Endo) group] to 51.7 ± 3.2%, confirming a contribution of NO or/and PGI_2_ in the relaxation of TFR. But the remained relaxation of TFR [the L-NAME + Indo + TFR (+Endo) group] is markedly stronger than TFR-induced relaxation in endothelium-denuded rat CBA [the TFR (−Endo) group] (*P* < 0.01); this suggests an involvement of another endothelial-derived mediator besides NO and PGI_2_, namely, a non-NO/PGI_2_ factor.

Figure 5 also shows that pretreatment of L-NAME + Indo did not affect TFR-induced relaxation in rat CBA without endothelium [the TFR (−Endo) group] (*P* > 0.01). The result is consistent with the characteristics of EDRFs.

### 3.5. Effect of PPG on TFR-Induced Non-NO/PGI_*2*_-Mediated Relaxation

**Figure 6:**
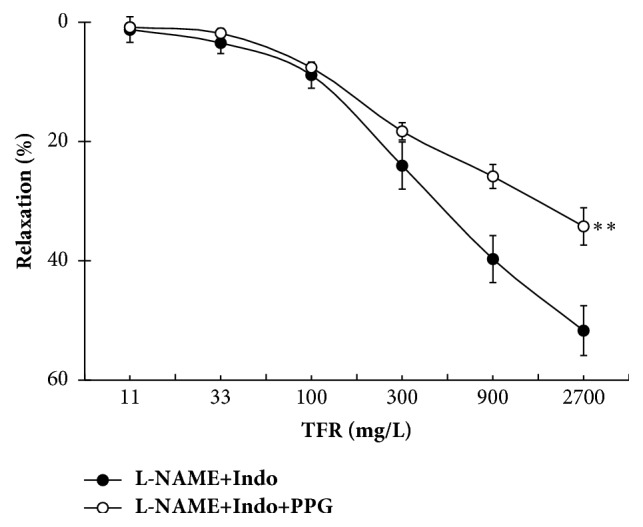
Effect of cystathionine-*γ*-lyase inhibitor DL-propargylglycine (PPG) on total flavones of* Rhododendron simsii *Planch flower- (TFR-) induced relaxation in endothelium-intact rat cerebral basilar artery in the presence of 30 *μ*M L-NAME and 10 *μ*M Indo (mean ± SD, *n* = 6). Pretreatment of 100 *μ*M PPG did not affect U_46619_-induced vessel tone (data not shown). ^*∗∗*^*P* < 0.01 versus the L-NAME + Indo group.

Pretreatment with 100 *μ*M PPG, an inhibitor of endogenous H_2_S-producing enzyme CSE, did not affect tone of the U_46619_-precontracted rat CBA but significantly inhibited TFR-evoked dilation in the presence of L-NAME + Indo (*P* < 0.01); *E*_max_ was reduced from 51.7 ± 3.2% in the L-NAME + Indo group to 39.4 ± 3.1% in the L-NAME + Indo + PPG group (Figure 6). Therefore, CSE-produced H_2_S may be engaged with the non-NO/PGI_2_-mediated relaxation of TFR in rat CBA.

### 3.6. Effect of TFR on the H_*2*_S Production

**Figure 7:**
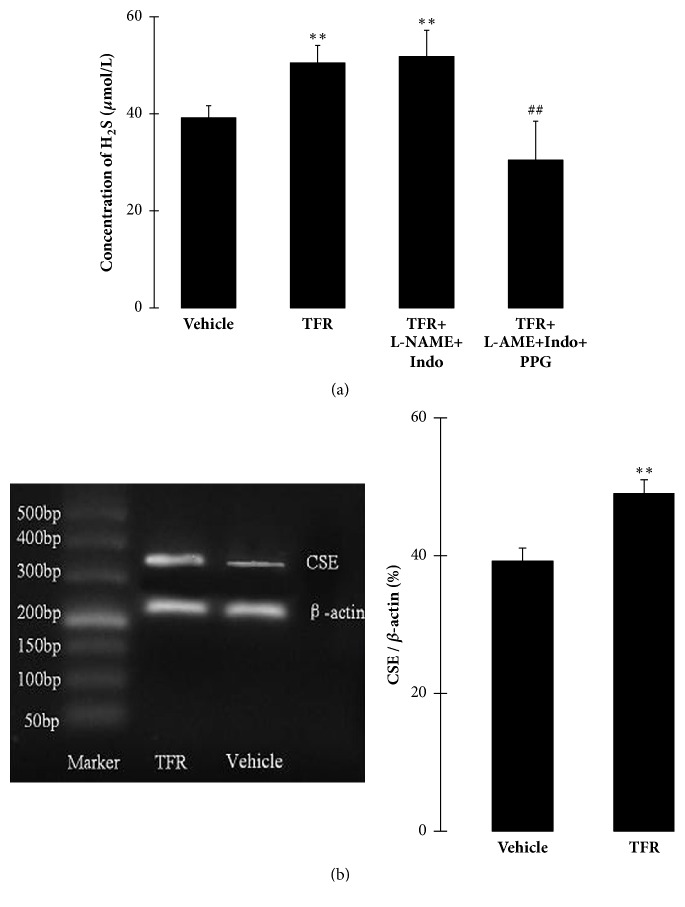
Effects of total flavones of* Rhododendron simsii *Planch flower (TFR) on H_2_S generation and cystathionine-*γ*-lyase (CSE) mRNA expression. (a) TFR-increased H_2_S production in luminal perfusate of endothelium-intact rat cerebral basilar artery and the effects of 30 *μ*M L-NAME, 10 *μ*M Indo and 100 *μ*M PPG on the increase (mean ± SD, *n* = 6). (b) Effects of TFR on CSE mRNA expression in primary cultured rat cerebral basilar artery endothelial cells (RT-PCR method, mean ± SD, *n* = 3). ^*∗∗*^*P* < 0.01 versus the vehicle group; ^##^*P* < 0.01 versus the TFR + L-NAME + Indo group.

Luminal perfusate was collected in the end of the vessel experiment, and H_2_S concentration was determined.  Figure 7(a) shows that the infusion of TFR (11 to 2700 mg/L) significantly promoted the H_2_S generation in endothelium-intact rat CBA; the H_2_S concentration was obviously increased from 39.2 ± 2.5 *μ*M in the vehicle group to 50.5 ± 3.6 *μ*M in the TFR group (*P* < 0.01).

Figure 7(a) also shows that the promoting effect of TFR on H_2_S production did not be markedly affected by co-pretreatment of L-NAME and Indo (*P* > 0.01), suggesting the promotion is resistant to L-NAME and Indo. But this promotion resistant to L-NAME and Indo could be abolished by CSE inhibitor PPG, and the H_2_S concentration was reduced from 51.8 ± 5.4 *μ*M in the TFR + L-NAME + Indo group to 30.5 ± 8.0 *μ*M in the TFR + L-NAME + Indo + PPG group (*P* < 0.01). The result suggests that neither NO nor PGI_2_, but the non-NO/PGI_2_ mechanism might involve the promotion of TFR on production of CSE-produced H_2_S.

### 3.7. Effect of TFR on CSE Expression in Rat CBA Endothelial Cells

The result of immunofluorescence staining confirmed that factor VIII, the endothelium marker, was unmistakably expressed in primary cultured rat CBA endothelial cells. RT-PCR analysis demonstrated that CSE mRNA is expressed in the cells. Compared with the vehicle group, 2700 mg/L TFR significantly increased the CSE mRNA expression in CBA endothelial cells (*P* < 0.01) (Figure 7(b)).

## 4. Discussion

In the present study, we have for the first time found that (1) CSE-produced H_2_S participated in the protective effect of TFR on cerebral I/R injury in rats and mice; (2) TFR induced a relaxation in rat CBA, which was mediated by EDHF and endothelial NO and/or PGI_2_; (3) CSE-produced H_2_S is involved in EDHF-mediated vasodilation of TFR in rat CBA, which contributed to the cerebral protection of TFR.

Cerebral infarct measurement is the gold standard for brain ischemic injury in an animal experiment. In the present study, it was found that the cerebral I/R-induced abnormal neurological symptom and cerebral infarct occurred more grievous in the CSE KO mice (CSE^−/−^ mice) than those in the wild type mice (CSE^+/+^ mice). In addition, the CSE knockdown by using the CSE-siRNA transfection in vivo resulted in an obvious aggravation of cerebral I/R injury in rat. These data demonstrated that CSE was involved in the cerebral I/R injury in mice and rats.

Previous studies demonstrated that TFR protected mouse brain and rat heart from I/R injury, respectively, in the dose range of 30~120 mg/kg (daily oral administration for 10 days) [17] and 25~100 mg/kg (intravenous injection) [22]. But the cerebral protective mechanism of TFR is still unclear and needs further investigation. In the present study, intravenous administration of 100 mg/kg TFR was used to study the mechanism. Protection of TFR 100 mg/kg was observed in the wild type mice and the control rats. However, the protective effect of TFR was lost in the CSE KO mice and was significantly weakened in the CSE knockdown rat. These results indicated that downregulation of CSE could attenuate the cerebral protection of TFR. CSE is an important endogenous H_2_S generating-enzyme. Its lack leads to a reduction of H_2_S production. Thus, these data suggest that CSE-produced H_2_S probably participated in the protective effect of TFR against cerebral I/R injury in mice and rats.

As aforementioned that CSE is selectively expressed in blood vessel, the present study focused on cerebral artery to investigate the role of CSE-produced H_2_S in the protection of TFR. It was found that, in the range of 11~2700 mg/L, TFR evoked a significant relaxation of endothelium-intact rat CBA with *E*_max_ of 73.4 ± 6.5%, which was markedly attenuated by the CSE-siRNA transfection, and indicated that CSE-produced H_2_S mediated the relaxation of TFR in rat CBA. Apparently, this cerebral vasorelaxation is beneficial for TFR protecting brain from I/R injury.

An attenuation of the relaxation of TFR in the CBA was observed after removing the endothelium. The result indicates that TFR induced an endothelium-dependent dilation in rat CBA and EDRFs were involved in the dilation. Endothelium-derived NO and PGI_2_, two classic EDRFs, play important roles in endothelium-dependent vasodilation. Some flavones could induce productions of NO and PGI_2_ in endothelial cells [23, 24]. In the present study, pretreatment with NO synthase inhibitor L-NAME, together with cyclooxygenase inhibitor Indo, significantly inhibited TFR-induced relaxation in endothelium-intact rat CBA. The result suggests the involvement of endothelial NO and/or PGI_2_ in relaxation of rat CBA to TFR. Meanwhile, it was observed that relaxation of endothelium-denuded rat CBA to TFR was still significant. The result suggests that TFR could likewise produce an endothelium-independent relaxation.

Endothelium-dependent relaxation cannot be fully explained by releases of NO and PGI_2_. EDHF is another notable mediator of vasodilation, which also contributes to endothelium-dependent vasorelaxation. EDHF-mediated relaxation is dependent on membrane hyperpolarization of VSMC and is resistant to NO synthase inhibitor and cyclooxygenase inhibitor. In the present study, relaxation of TFR in endothelium-intact rat CBA was significantly attenuated in the presence of L-NAME and Indo, but the remnant relaxation is significantly stronger than relaxation of endothelium-denuded rat CBA to TFR, the TFR-induced endothelium-independent relaxation. Obviously, difference between this remnant relaxation and the endothelium-independent relaxation is a non-NO/PGI_2_ relaxation.

As we well know, non-NO/PGI_2_ relaxation is an essential trait of EDHF. K_Ca_ channel is involved in EDHF-mediated relaxation [25], and K_Ca_ channel blockers, such as tetraethylammonium, could inhibit this relaxation [26]. Our previous study shows that TFR could induce a non-NO/PGI_2_-mediated hyperpolarization in VSMC of the CBA from rat subjected to cerebral I/R injury, which was almost abolished by tetraethylammonium [27]. Thus, it could be concluded that the TFR-induced non-NO/PGI_2_ relaxation in rat CBA is an EDHF response.

Unlike NO and PGI_2_, the nature of EDHF has not been definitively identified, especially in cerebral arteries. H_2_S, a gaseous signal molecule, is known to be released from endothelium [28]. Numerous studies indicated that the concentrations of H_2_S ranged within 30~300 *μ*mol/L in blood vessels as well as in many other tissues including the heart, brain, and kidney [29–32]. However, it was observed that a severe cardiac contractility depression in rat was observed when H_2_S donor NaHS was infused to increase blood gaseous H_2_S at 7 *μ*M [33]. This contradiction may be due to that the gaseous H_2_S in rat blood is relatively low, and about 80% of H_2_S exists as HS^−^. So far the physiological level and the toxic level of H_2_S are still not ascertained, but no evidence shows that endogenous H_2_S could produce significant toxicity in vivo. H_2_S is regarded as EDHF because hyperpolarization is virtually abolished in the blood vessels of CSE-deleted mice [34]. In our previous studies, EDHF-mediated hyperpolarization of VSMC and vasodilation in rat MCA were abolished by H_2_S generating-enzyme CSE inhibitor PPG [12, 13]. In the present study, non-NO/PGI_2_-mediated relaxation, namely, EDHF-mediated relaxation induced by TFR in rat CBA, was significantly inhibited by 100 *μ*mol/L PPG, suggesting an involvement of CSE-produced H_2_S in EDHF-mediated relaxation of TFR. This is in agreement with the previous study that PPG could obviously attenuate EDHF-mediated hyperpolarization and vasorelaxation of rat CBA to hyperin, a primary chemical constituent in TFR [15].

CSE is a predominant H_2_S generating-enzyme in heart and vascular tissues. Immunohistological examination indicates that the predominant localization of CSE protein is in the endothelial layer of vascular tissues in mice. The present study for the first time found that CSE mRNA is present in rat CBA endothelial cells, and TFR significantly increased the CSE mRNA expression. The RhoA/Rho-kinase signaling pathway is a promising therapeutic target, and it could downregulate expression and activation of endothelial NO synthase as well as NO production [35]. Similar to NO, endothelial CSE-produced H_2_S has also been identified as an EDRF. A previous study showed that TFR could inhibit RhoA/Rho-kinase pathway [36]. Hence, it is possible that TFR-increased CSE expression may be due to the RhoA/Rho-kinase pathway inhibition. But this deduction needs more data to be confirmed in future study. In addition, TFR significantly promoted H_2_S production in endothelium-intact rat CBA, and this promotion was not affected by L-NAME plus Indo, indicating that neither NO nor PGI_2_ was involved with TFR-promoted H_2_S generation. However, the promotion of TFR resistant to L-NAME and Indo could be inhibited by CSE inhibitor PPG. These data suggest that TFR could increase CSE-produced H_2_S in endothelium-intact rat CBA via a non-NO/PGI_2_ mechanism. This is in consistence with the aforementioned involvement of CSE-produced H_2_S in the EDHF-mediated relaxation of TFR. H_2_S could activate K_Ca_ channel to cause vasorelaxation in rat cerebral artery [14]. Perhaps the EDHF-mediated relaxation is due to TFR releasing CSE-produced H_2_S to activate K_Ca_ channel in rat CBA.

Because CSE is mainly expressed in vascular endothelial cells and VSMCs, the above-mentioned involvement of CSE-produced H_2_S in the protection of TFR against cerebral I/R injury can be explained by the contribution of the cerebral vasorelaxation, or rather that TFR protects brain from I/R injury via CSE-produced H_2_S causing a cerebrovascular relaxation. However, flavones have other biological activities such as anti-inflammatory and anti-reactive oxygen species (ROS). These activities are beneficial for cerebral protection. H_2_S has ROS scavenging effect [37], and TFR could increase endogenous CSE-produced H_2_S. Thus, perhaps more than one mechanism is involved in the cerebral protection of TFR, but the vasodilation is one of important mechanisms.

Ischemic preconditioning (IPC) can initiate endogenous mechanism to protect the brain from I/R injury [38]. Morphine and some agents could mimic IPC to exert neuroprotection [39]. In the present study, TFR pretreatment was used to induce a pharmacological preconditioning against cerebral I/R injury. IPC-induced protection appears in a time-dependent manner. Early protection is presented for 3~4 h immediately after IPC, and delayed protection reappears after 12~24 h and maintains for 48~72 h. There is a protection-free interval between early and delayed protection. Combining with our previous study that TFR could produce both early and delayed protection against rat myocardial I/R injury [40], TFR pretreatment probably produce a pharmacological preconditioning to attenuate cerebral postischemic brain injury.

A posttreatment against I/R injury is more important in the clinic. At present, efficacious treatment approved for ischemic stroke is thrombolysis, but only a small number of patients can be suitable for this treatment. It is necessary to develop an efficacious treatment for ischemic stroke. Cerebral relaxation of TFR could lead to an increase in cerebral blood flow, which is beneficial for brain repair and functional recovery after ischemic stroke. Moreover, some flavones such as puerarin were found to inhibit infarct formation in postischemic brain in rat [41]. Therefore, TFR has a potential application for the posttreatment against cerebral I/R injury, but this has yet to be investigated.

## 5. Conclusions

The present study for the first time provided experimental data to demonstrate the role of CSE-produced H_2_S in the protective effect of TFR against cerebral I/R injury. The cerebral protection of TFR was engaged with CSE-produced H_2_S, and TFR could relax rat CBA in an endothelium-dependent manner, which was mediated by EDHF as well as NO and/or PGI_2_. CSE-produced H_2_S participated in the EDHF-mediated relaxation of TFR in rat CBA. Thus, CSE-produced H_2_S participated the protective effect of TFR on rat cerebral I/R injury via relaxing cerebral artery. These findings are very useful to illustrate the mechanism of* Rhododendron simsii *Planch flower treating ischemic cerebral apoplexy.

## Data Availability

The data used to support the findings of this study are available from the corresponding author upon request.
